# Silymarin Supplementation in Active Rheumatoid Arthritis: Outcomes of a Pilot Randomized Controlled Clinical Study

**DOI:** 10.3390/medicina60060999

**Published:** 2024-06-18

**Authors:** Georgeta Stefanovici Zugravu, Carmen Pintilescu, Carmen-Marinela Cumpat, Sorin Dan Miron, Anca Miron

**Affiliations:** 1 Faculty of Pharmacy, Grigore T. Popa University of Medicine and Pharmacy, 16, Universitatii Street, 700115 Iasi, Romania; 2 Clinical Rehabilitation Hospital, 14, Pantelimon Halipa Street, 700661 Iasi, Romania; 3 Faculty of Economics and Business Administration, Alexandru Ioan Cuza University of Iasi, 22, Carol I Boulevard, 700505 Iasi, Romania; 4 Faculty of Medicine, Grigore T. Popa University of Medicine and Pharmacy, 16, Universitatii Street, 700115 Iasi, Romania

**Keywords:** rheumatoid arthritis, silymarin, functional status, fatigue, pain, depression

## Abstract

*Background and Objectives*: Coadministration of natural products to enhance the potency of conventional antirheumatic treatment is of high interest. This study aimed to assess the impact of administration of silymarin (a nutritional supplement) in patients with active rheumatoid arthritis under treatment with conventional disease-modifying antirheumatic drugs. *Materials and Methods*: One-hundred and twenty-two patients diagnosed with active rheumatoid arthritis and treated with conventional disease-modifying antirheumatic drugs were randomly assigned to either control or intervention groups; the latter was supplemented with silymarin (300 mg/day) for 8 weeks. Indicators of disease activity, inflammatory markers, disease activity and disability indices, European League Against Rheumatism responses, fatigue, depression, and anxiety scores were determined at baseline and week 8. *Results*: Silymarin supplementation significantly reduced the number of tender and swollen joints, duration of morning stiffness, severity of pain, disease activity and disability indices, European League Against Rheumatism responses, levels of fatigue, depression, and anxiety. According to our results, silymarin substantially improved patients’ general condition. *Conclusions*: Our study provides evidence for the benefits of silymarin supplementation to disease-modifying antirheumatic drugs in patients with active rheumatoid arthritis.

## 1. Introduction

Rheumatoid arthritis (RA) is one of the most common chronic inflammatory diseases, affecting 0.1–2% of the world’s population [1,2]. Inappropriate management of the disease results in permanent functional disability with significant individual and socioeconomic burdens [2]. The therapy, including non-steroidal anti-inflammatory drugs (NSAIDs), glucocorticoids, conventional synthetic, biological, and targeted synthetic disease-modifying antirheumatic drugs (DMARDs), aims at reducing symptoms, delaying disease progression, preventing complications, and enhancing life quality [2,3,4]. The above-mentioned drugs have side effects and/or higher costs, and therefore, coadministration of natural products to enhance their bioactivity and reduce their toxicity is a promising therapeutic approach.

Medicinal plants have been used to treat arthritis for centuries [5]. Choudhary et al. carried out a vast and detailed bibliographic investigation and found 485 plant species traditionally used in the treatment of arthritis all over the world [5]. Plants contain compounds belonging to different chemical classes (phenolics, flavonoids, coumarins, terpenoids, and alkaloids) that have the potential to attenuate inflammation by various mechanisms (reduction in inflammatory response, oxidative stress and angiogenesis, immunoregulation, regulation of microRNAs, and fibroblast-like synoviocytes) [6,7]. Moreover, in various arthritic models, the combination of plant extracts/pure phytochemicals with DMARDs considerably improved the antiarthritic activity of the latter and attenuated the adverse effects characteristic of DMARDs [6]. 

Silymarin is a complex mixture of flavonolignans (60–80%) and other flavonoids (taxifolin) isolated from the fruits of *Silybum marianum* (L.) Gaertn. (milk thistle). Silybin (silibinin) is the major flavonolignan, representing 50–70% of silymarin [8,9]. Silymarin has excellent liver protective and regenerative effects and is commercialized as an herbal medication or nutritional supplement. Its main indications are viral- and toxin-induced hepatitis, alcoholic and non-alcoholic fatty liver disease, cirrhosis, *Amanita* mushroom poisoning, and hypercholesterolemia [10,11,12]. In addition, recent clinical studies have reported the efficacy of silymarin and/or silibinin in diabetes [13], cardiometabolic syndrome [14], Alzheimer’s disease [15], melasma [16,17], and as radioprotective agents [18]. Silymarin has a good safety profile, and no significant toxicity has been reported in human studies [11]. 

The present study was designed to assess the impact of silymarin supplementation on patients with active RA under treatment with conventional DMARDs. In this respect, RA-specific variables (clinical parameters, biochemical markers, disease activity indices, disability index, and European League Against Rheumatism (EULAR) responses) and the level of common comorbidities (fatigue, depression, and anxiety) were evaluated. The correlations between disease severity, functional status, and the above-mentioned comorbidities were also investigated. 

## 2. Materials and Methods

### 2.1. Participants

A total of 179 patients (153 women, 26 men), diagnosed with RA according to the American College of Rheumatology (ACR)/EULAR 2010 criteria [19], were randomly recruited and evaluated at the Clinical Rehabilitation Hospital (Rehabilitation, Physical Medicine and Balneology Section), Iasi, Romania, from April to November 2022. Twenty-one patients did not meet the inclusion criteria; 32 patients had one or more exclusion criteria, and 4 patients refused to be enrolled in this study. Finally, this study included 122 patients (103 women and 19 men). Data on age, height, weight, body mass index (BMI), comorbidities, disease duration and stage, functional capacity, gender, and residency were collected at baseline; disease stage and functional capacity were evaluated according to previous reports [20,21]. Patients enrolled were under treatment with conventional DMARDs (methotrexate, leflunomide, sulfasalazine, hydroxychloroquine, and azathioprine) either as monotherapy or combination therapy (two- or three-drug combination). Other inclusion criteria were as follows: disease activity score in 28 joints (DAS28) calculated with C reactive protein (CRP) higher than 3.2 (active RA); cessation of administration of steroids and NSAIDs at least one month before beginning this study; and signing of written informed consent. Medical conditions such as sepsis, abscesses, active tuberculosis or any other active infection, malignancy, history of severe and uncontrolled cardiac, renal, and mental disorders, history/presence of other inflammatory autoimmune diseases, therapy with biological DMARDs (tumor necrosis factor-α (TNF-α) and interleukin (IL)-6, -17 inhibitors), targeted synthetic DMARDs (Janus kinase inhibitors), and metronidazole, pregnancy, lactation, alcohol or substance abuse, use of oral contraceptive pills, consumption of dietary supplements and herbal teas with anti-inflammatory and antioxidant effects were considered exclusion criteria. In addition, all participants were instructed to eat a balanced diet and to abstain from plant products (fruits, vegetables) in excess over this 8-week study. 

### 2.2. Study Design

This pilot randomized controlled trial followed the rules of the Declaration of Helsinki of 1975/83 and was approved by the Research Ethics Committee of Grigore T. Popa University of Medicine and Pharmacy Iasi, Romania (approval ID: 68/13.04.2021) and the Ethics Committee of the Clinical Rehabilitation Hospital Iasi, Romania (approval ID: 9/18.04.2022). Patients meeting the inclusion criteria (*n* = 122) were randomly assigned to either the intervention or control groups (1:1 ratio). All patients were under treatment with conventional DMARDs. Patients in the intervention group were supplemented with Silymarin Forte (a nutritional supplement produced by Zenyth Pharmaceuticals SRL, Neamt, Romania), one capsule daily for 8 weeks; each capsule contained 375 mg of standardized extract obtained from milk thistle fruits equivalent to 300 mg of silymarin. 

### 2.3. Outcomes

The primary objective was to evaluate the impact of silymarin supplementation on clinical outcomes, disease activity, and physical function in patients with active RA; the secondary outcomes included improvements in common comorbidities (fatigue, depression, and anxiety).

### 2.4. Patients’ Evaluation

At baseline and week 8, the following variables were evaluated: tender and swollen joint count in 28 joints (TJC28 and SJC28, respectively), morning stiffness (in min.), patient’s assessment of pain, patient’s global assessment of disease activity (PtGA), physician’s global assessment of disease activity (PhGA), erythrocyte sedimentation rate (ESR), and CRP. Patient’s assessment of pain and PhGA were evaluated on a visual analogue scale (VAS) ranging from 0 to 10 cm (0 = no pain, 10 = severe pain, and 0 = best, 10 = worst, respectively) [22,23]. PtGA was determined on a VAS from 0 to 100 mm (0—best; 100—worst) [24]. ESR was determined by the Westergren method (THERMA analyzer, Linear Chemicals, Montgat, Barcelona, Spain). Serum CRP was determined by immunoturbidimetry (biochemical analyzer XL 1000, Erba, Lachema S.R.O., Brno, Czech Republic).

The disease activity was evaluated at the beginning and end of this study by several composite scores: DAS28, the simplified disease activity index (SDAI), and the clinical disease activity index (CDAI). DAS28 was calculated using TJC28, SJC28, PtGA, and a marker of acute inflammation, ESR (DAS28-ESR) or CRP (DAS28-CRP) [24]. SDAI was calculated as the sum of TJC28, SJC28, PtGA, PhGA, and CRP levels, whereas CDAI calculation included only the first four parameters, excluding CRP [22]. The patients’ functional status was evaluated at baseline and week 8 on the basis of the health assessment questionnaire-disability index (HAQ-DI) which estimates the patients’ ability to perform 20 daily life activities [25]. EULAR responses, EULAR-ESR and EULAR-CRP, were assessed based on the improvement in DAS28-ESR and DAS28-CRP scores, respectively [22,24].

This study also evaluated the impact of silymarin supplementation on fatigue, depression, and anxiety. Fatigue was evaluated using a VAS (0–100 mm) [26]. Depression was assessed by a 21-item Beck Depression Inventory scale II (BDI-II) questionnaire [27,28]. The 7-item generalized anxiety disorder (GAD-7) scale [29] was used to detect anxiety and evaluate its severity. 

### 2.5. Statistical Analysis

The statistical analysis was performed with SPSS software (version 25). The normality of the data distribution was evaluated by the Kolmogorov–Smirnov test. The independent samples *t*-test was used to compare the differences between the intervention and control groups, while the paired samples *t*-test was applied for within-group comparisons. The associations between qualitative variables and those between patient groups were evaluated by a Chi-Square test. Values of *p* less than or equal to 0.05 were considered statistically significant. The two-step cluster analysis was used in studying patients in the intervention and control groups, taking into consideration both qualitative and quantitative variables. The correlations between disease activity and disability indices (DAS28-ESR, DAS28-CRP, SDAI, CDAI, and HAQ-DI, respectively) and levels of fatigue, depression, and anxiety (VAS-F, BDI-II, and GAD-7, respectively) at week 8 were evaluated using the Pearson’s correlation coefficient. 

## 3. Results

### 3.1. Patients’ Characteristics

**Figure 1:**
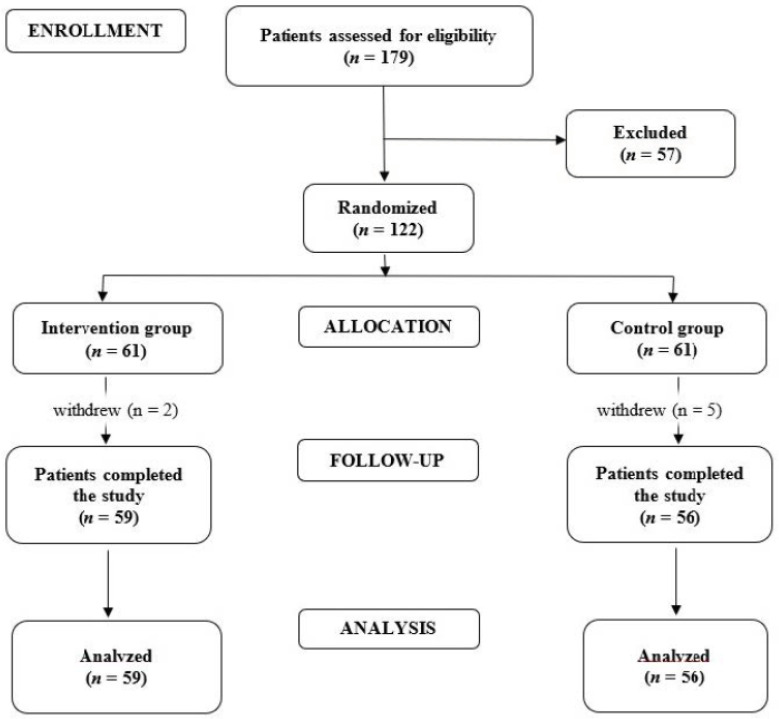
Flow diagram of this study.

A total of 179 patients diagnosed with RA were evaluated initially, of which 122 were enrolled and randomized to the control and intervention groups (*n* = 61 in each group) (Figure 1). 

The control and intervention groups did not differ significantly (*p* > 0.05) with respect to age, gender, residence, height, weight, BMI, smoking status, comorbidities, disease duration and stage, and functional capacity. RF, a disease marker for RA, was not significantly different (*p* > 0.05) between the two groups (Table 1).

### 3.2. Indicators of Disease Activity

As shown in Table 2, the two groups were significantly different at baseline with respect to pain intensity, PtGA, and PhGA (*p* < 0.05); there were no significant differences in baseline levels of TJC28, SJC28, and morning stiffness (*p* > 0.05). At week 8, in the intervention group, all indicators of disease activity were significantly reduced compared to their baseline values (*p* ≤ 0.05). In the control group, except PhGA (*p* = 0.051), the scores significantly increased (*p* ≤ 0.05) indicating that the patients’ condition worsened. In addition, at week 8, all indicators of disease activity were significantly improved in the intervention group compared to the control group (*p* = 0.000). 

### 3.3. Inflammatory Markers

There were no significant differences in inflammatory markers (ESR, CRP) between the two groups at baseline (*p* > 0.05). At week 8, in the intervention group, ESR was reduced while CRP increased compared with their baseline values, but the changes were not statistically significant (*p* > 0.05). In the control group, both markers decreased, with no significant differences between values at baseline and week 8 (*p* > 0.05). In addition, ESR and CRP levels were not significantly different between the two groups at the end of this study (*p* > 0.05) (Table 3). 

### 3.4. Disease Activity Indices

**Figure 2:**
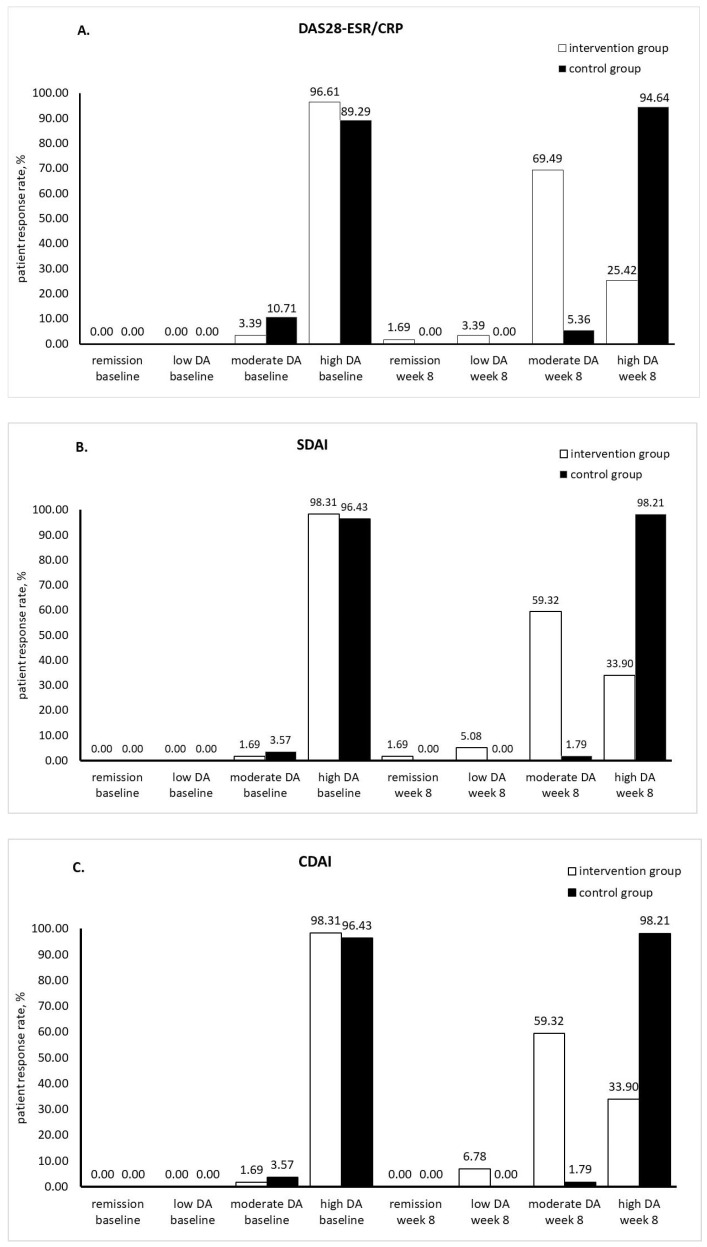
Differences in disease activity indices between the intervention and control groups ((**A**). DAS28-ESR/CRP, (**B**). SDAI, (**C**). CDAI; DA: disease activity; DAS28-ESR: disease activity score in 28 joints calculated with erythrocyte sedimentation rate; DAS28-CRP: disease activity score in 28 joints calculated with C reactive protein; SDAI: simplified disease activity index; CDAI: clinical disease activity index).

According to disease activity indices, high disease activity is characterized by DAS28-ESR/CRP > 5.1, SDAI > 26, CDAI > 22, moderate activity by 3.2 < DAS28-ESR/CRP ≤ 5.1, 11 < SDAI ≤ 26, 10 < CDAI ≤ 22, low activity by 2.6 < DAS28-ESR/CRP ≤ 3.2, 3.3 < SDAI ≤ 11, 2.8 < CDAI ≤ 10, and remission by DAS28-ESR/CRP ≤ 2.6, SDAI ≤ 3.3, CDAI ≤ 2.8 [22]. According with the mean scores of disease activity indices, at baseline, patients in both groups had high disease activity with no significant differences between groups (*p* > 0.05). At week 8, patients in the intervention group had moderate disease activity, whereas those in the control group still had high disease activity with elevated disease activity indices in comparison with baseline, with the increase in CDAI being statistically significant (*p* < 0.05). At week 8, the disease activity indices were significantly different between groups (*p* = 0.000) (Table 4). The percentages of patients having different levels of disease activity in each group, at baseline and week 8, are illustrated in Figure 2. According to DAS28-ESR/CRP, at baseline, 3.39% and 96.61% of patients in the intervention group had moderate and high disease activity, respectively. After 8-week silymarin supplementation, the percentage of high disease activity patients dramatically declined to 25.42%; the other patients in the intervention group showed remission, low and moderate disease activity (1.69%, 3.39% and 69.49%, respectively). After 8 weeks, in the control group, patients with moderate disease activity diminished from 10.71% to 5.36%, whereas those with high disease activity increased from 89.29% to 94.64% (Figure 2A). Similarly, according to SDAI and CDAI, the percentage of high disease activity patients showed a significant failure from 98.31% to 33.90% after 8-week silymarin supplementation. A modest increase in patients having high disease activity was noticed in the control group (96.43% at baseline vs. 98.21% at week 8) (Figure 2B,C).

### 3.5. Disability Index

**Figure 3:**
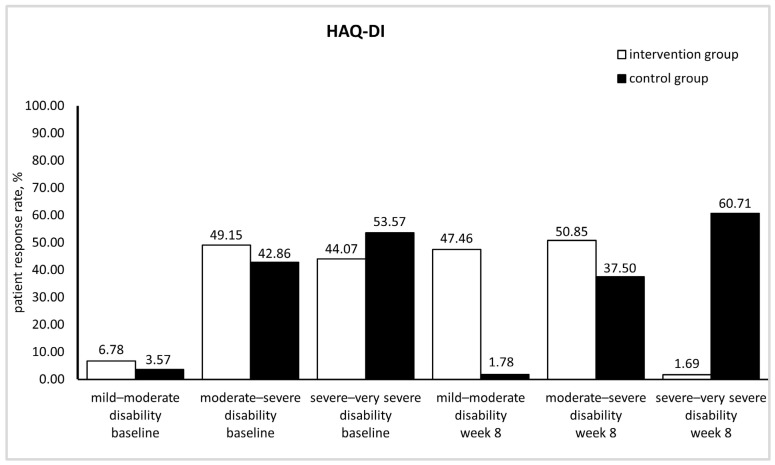
Differences in disability index between the intervention and control groups (HAQ-DI: health assessment questionnaire-disability index).

HAQ-DI values of 0–1, 1–2, and 2–3 represent mild to moderate, moderate to severe, and severe to very severe disability, respectively [30]. According to mean HAQ-DI values at baseline, patients in the control and intervention groups had moderate to severe disability; HAQ-DI scores were not significantly different between groups (*p* > 0.05). At the end of this study, patients in the intervention group still had moderate to severe disability, but HAQ-DI value significantly decreased in comparison with baseline (*p* < 0.05). In contrast, in the control group, disability became worse; the HAQ-DI score significantly increased in comparison with baseline (*p* < 0.05) indicating severe to very severe disability (Table 4). Figure 3 clearly illustrates the positive impact of silymarin supplementation on patients’ mobility. Under silymarin supplementation, the percentage of patients having moderate to severe disability was slightly elevated (from 49.15% to 50.85%), whereas patients with mild to moderate disability rose from 6.78% to 47.46%. Concomitantly, patients with severe to very severe disability drastically declined from 44.07% to 1.69%. On the other hand, over the 8-week trial, in the control group, the percentages of patients with mild to moderate and moderate to severe disability dropped (from 3.57% to 1.78% and from 42.86% to 37.50%, respectively), while those with severe to very severe disability augmented (from 53.57% to 60.71%). 

### 3.6. EULAR Responses

**Figure 4:**
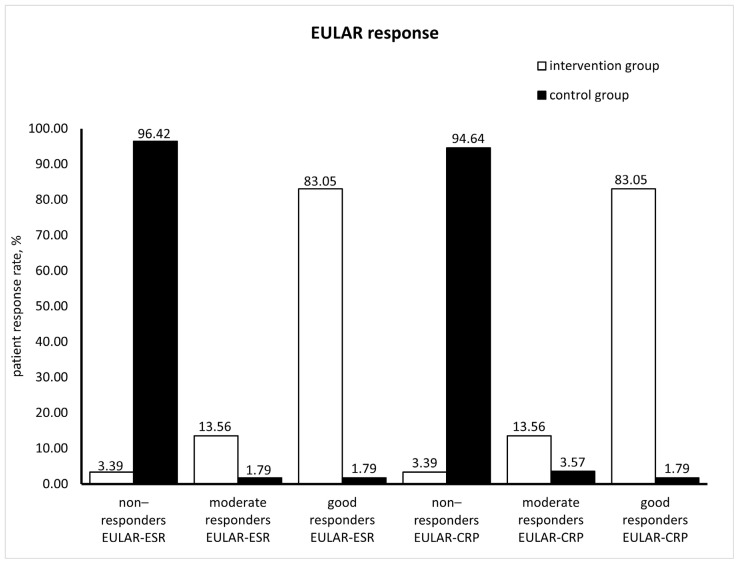
EULAR responses in the intervention and control groups (EULAR-ESR: European League Against Rheumatism response calculated with erythrocyte sedimentation rate; EULAR-CRP: European League Against Rheumatism response calculated with C reactive protein).

According to EULAR response criteria, patients are good (DAS28 improvement > 1.2 and actual DAS28 ≤ 3.2), moderate (DAS28 improvement > 0.6 and ≤1.2 and actual DAS28 ≤ 3.2/actual DAS28 > 3.2 and ≤5.1; DAS28 improvement > 1.2 and actual DAS28 > 3.2 and ≤5.1/actual DAS28 > 5.1) or non-responders (DAS28 improvement > 0.6 and ≤1.2 and actual DAS28 > 5.1; DAS28 improvement ≤ 0.6 and actual DAS28 ≤ 3.2/actual DAS28 > 3.2 and ≤5.1/actual DAS28 > 5.1) [22]. Data on EULAR responses are shown in Figure 4. After 8-week supplementation with silymarin, 83.05% of the patients in the intervention group had good EULAR-ESR and EULAR-CRP responses. In the control group, according to EULAR-ESR and EULAR-CRP scores, 96.42% and 94.64% of patients were non-responders. 

### 3.7. Fatigue, Depression, and Anxiety

**Figure 5:**
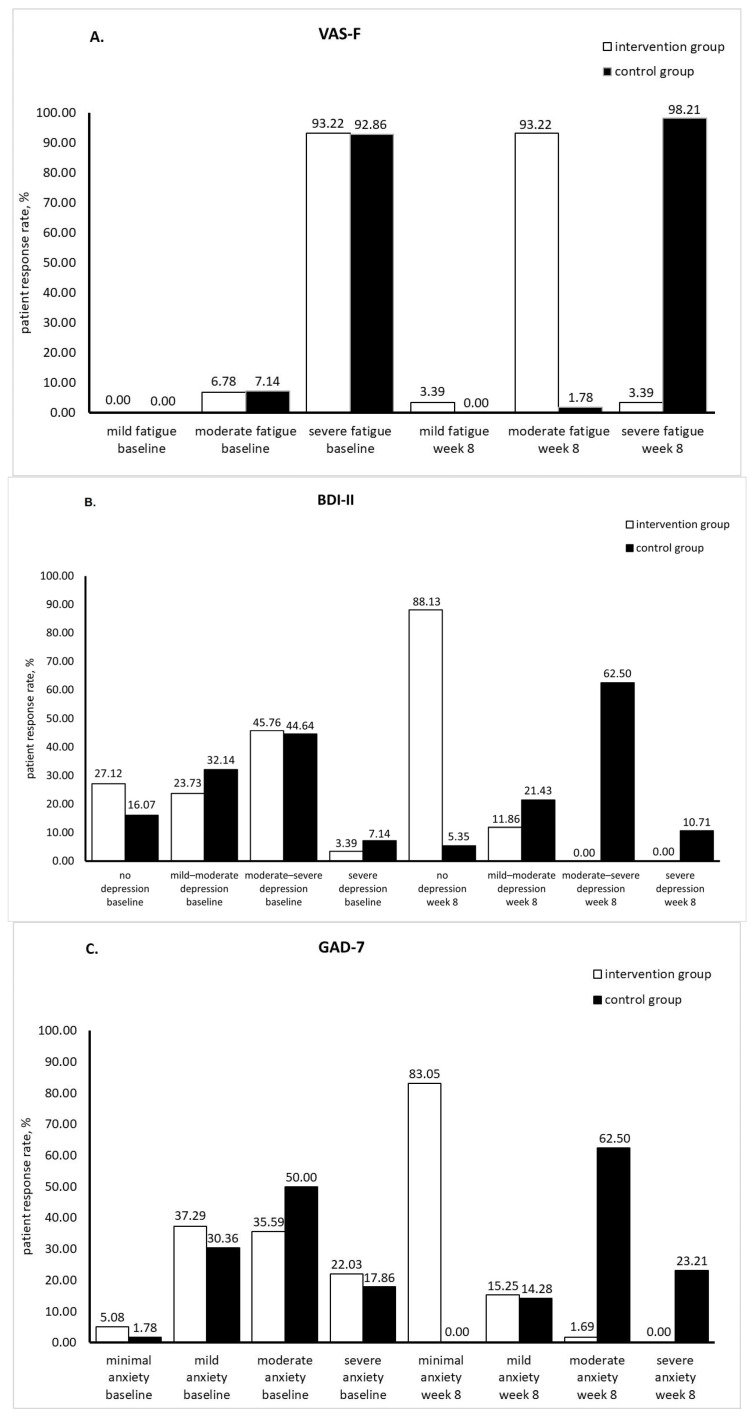
Fatigue (**A**), depression (**B**), and anxiety (**C**) scores at baseline and week 8 (VAS-F: visual analogue scale-fatigue; BDI-II: Beck depression inventory II; GAD-7: generalized anxiety disorder-7).

Fatigue was evaluated as mild, moderate, or severe depending on VAS scores (<20, 20–50, and >50, respectively) [31,32]. Depression was assessed depending on BDI-II score as follows: no depression (0–13), mild to moderate depression (14–19), moderate to severe depression (20–28), and severe depression (29–63) [28]. Anxiety level was indicated by GAD-7 score: minimal, mild, moderate, and severe anxiety for GAD-7 scores of 0–4, 5–9, 10–14, and 15–21, respectively [29]. At baseline, there were no significant differences in mean values of fatigue, BDI-II, and GAD-7 scores between patient groups (*p* > 0.05). Patients in both groups reported severe fatigue, mild to moderate depression, and moderate anxiety. After 8-week supplementation with silymarin, patients’ condition in the intervention group markedly improved. Fatigue, BDI-II, and GAD-7 scores significantly decreased compared to their baseline values (*p* = 0.000). According to scores at week 8, patients in the intervention group had moderate fatigue, no depression, and minimal anxiety. In contrast, in the control group, fatigue, BDI-II, and GAD-7 scores significantly increased compared to baseline (*p* < 0.05) indicating deterioration in the patients’ condition (Table 5). The benefits of silymarin supplementation on fatigue, depression, and anxiety are depicted in Figure 5. Under supplementation with silymarin, the percentage of patients having severe fatigue decreased from 93.22% (baseline) to 3.39% (week 8). In the control group, patients reporting severe fatigue increased from 92.86% (baseline) to 98.21% (week 8) (Figure 5A). Similarly, silymarin supplementation attenuated depression and anxiety in RA patients. In the intervention group, the percentage of patients reporting depression decreased from baseline to week 8 while patients reporting no depression markedly increased from 27.12% (baseline) to 88.13% (week 8). Throughout this study, in the control group, there was an increase in patients with moderate to severe depression and severe depression (from 44.64% to 62.50% and from 7.14% to 10.71%, respectively) (Figure 5B). Patients with minimal anxiety showed an impressive increase in the intervention group (from 5.08% at baseline to 83.05% at week 8), while the number of patients reporting mild, moderate, or severe anxiety decreased from the start to the end of this study. The control group was characterized by an increase in patients with moderate and severe anxiety (from 50.00% to 62.50% and from 17.86% to 23.21%, respectively) (Figure 5C). 

### 3.8. Impact of Silymarin Supplementation on Patients’ General Condition

**Figure 6:**
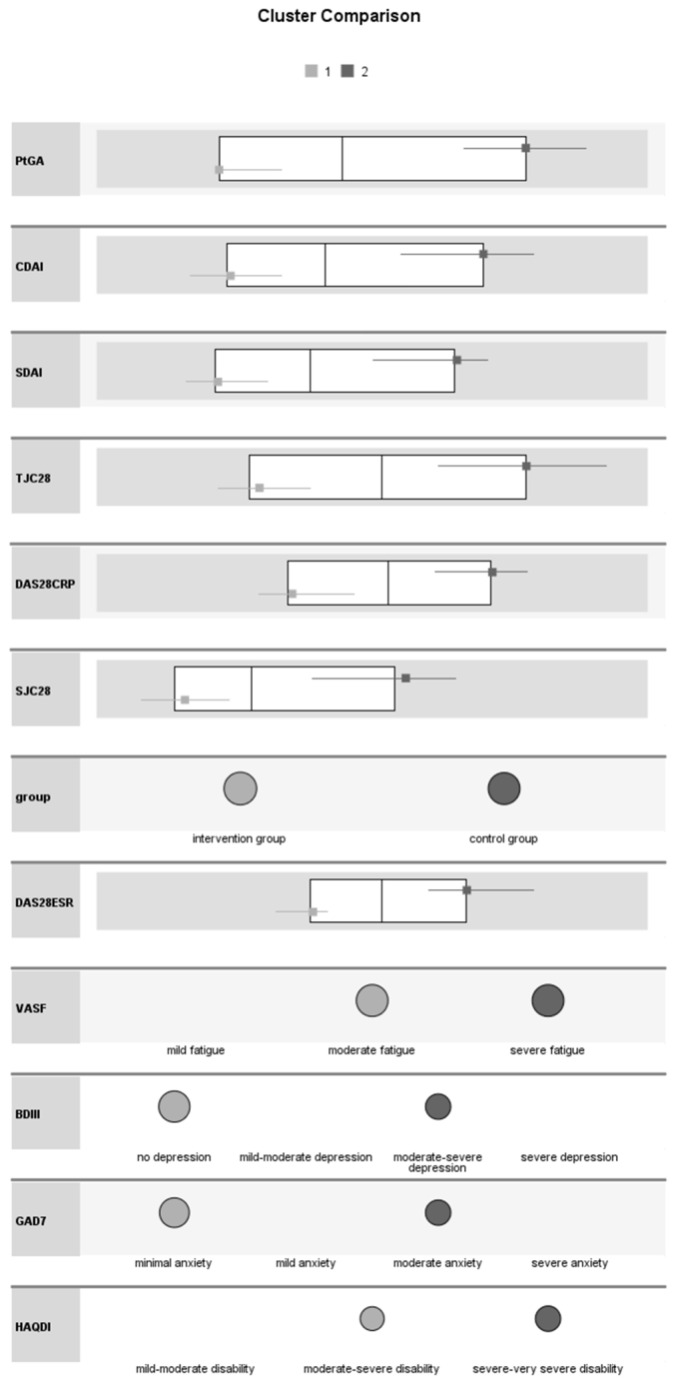
Comparative analysis of patients’ general condition in the intervention and control groups at week 8 (PtGA: patient’s global assessment of disease activity; CDAI: clinical disease activity index; SDAI: simplified disease activity index; TJC28: tender joint count in 28 joints; DAS28-CRP: disease activity score in 28 joints calculated with C reactive protein; SJC28: swollen joint count in 28 joints; DAS28-ESR: disease activity score in 28 joints calculated with erythrocyte sedimentation rate; VAS-F: visual analogue scale-fatigue; BDI-II: Beck depression inventory II; GAD-7: generalized anxiety scale-7; HAQ-DI: health assessment questionnaire-disability index).

At week 8, patients in the intervention group had a substantial improvement in their general condition (lower disease activity according to self-assessment, smaller proportion of tender and swollen joints in 28 joints, lower disease activity and disability indices, lower fatigue, depression, and anxiety scores compared to the control group) (Figure 6).

### 3.9. Correlations between Disease Severity, Functional Status and Levels of Fatigue, Depression and Anxiety

According to Pearson’s correlation coefficient values (around 0.3, 0.5, and >0.7), correlations are weak, moderate, and strong, respectively [33]. At week 8, in the intervention group, there were strong correlations between disease activity indices (DAS28-ESR, DAS28-CRP, SDAI, CDAI) and weak-to-moderate correlations between disease activity indices and HAQ-DI. Disease activity indices were moderately associated with VAS-F and weakly associated with BDI-II and GAD-7. On the other hand, HAQ-DI was weakly to moderately associated with VAS-F, BDI-II, and GAD-7. The aforementioned correlations were statistically significant (*p* < 0.05) except for the correlations between disease activity indices and BDI-II, and disease activity indices and GAD-7. At week 8, in the control group, all indices (DAS28-ESR, DAS28-CRP, SDAI, CDAI, HAQ-DI) were weakly associated with VAS-F, BDI-II, and GAD-7 (Table 6). 

## 4. Discussion

This study evaluated the efficacy of silymarin as adjunctive therapy in RA. Silymarin significantly improved the disease activity indices, disability index, and EULAR responses in RA patients under treatment with conventional DMARDs compared with those in the control group receiving only conventional DMARDs. These effects are undoubtedly related to the anti-inflammatory and antioxidant potential of silymarin. Silymarin has been reported to attenuate inflammation in various experimental models, both in vitro [34] and in vivo models (carrageenan and papaya latex-induced rat paw edema, arachidonic acid-induced mouse ear edema, mycobacterial adjuvant-induced arthritis, Freund’s adjuvant-induced arthritis, and monoiodoacetate-induced osteoarthritis rat models) [35,36,37,38]. Previous clinical studies investigated the effects of silymarin on patients with knee osteoarthritis. According to the Knee Injury and Osteoarthritis Outcome Score (KOOS) system, which assesses five outcomes (pain, symptoms, daily activities, sport/recreation, and life quality), silymarin (300 mg/day, 8 weeks) exerted analgesic and anti-inflammatory effects, causing greater score improvement compared to meloxicam (15 mg/day, 8 weeks) or piroxicam (20 mg/day, 8 weeks). When combined with meloxicam or piroxicam, silymarin potentiated their activity [39,40]. Two clinical studies conducted by Shavandi et al. evaluated the effects of silymarin supplementation (140 mg × 3/day, 3 months) in RA patients under standard treatment (methotrexate, hydroxychloroquine, sulfasalazine, azathioprine, prednisolone, NSAIDs, alendronate, and calcium supplements). The authors reported a significant decrease in the DAS28 score but no significant impact on seric IL-1ß and TNF-α after 3-month silymarin supplementation compared to baseline values [41,42]. Both clinical studies conducted by Shavandi et al. were single-arm trials that evaluated the effects of silymarin supplementation over time (baseline vs. month 3). In contrast to these two studies, our study was a two-arm randomized trial assessing the effects of silymarin supplementation in comparison with a control group (not supplemented with silymarin). Furthermore, our study enrolled only patients with active RA (DAS28-CRP > 6.0) and disease duration for more than 5 years. In our study, despite the remarkable improvement in disease activity and disability indices and EULAR responses, silymarin supplementation did not significantly impact ESR and CRP levels in comparison with baseline and control values. This result might be attributed to the size of patient groups and the duration of this study. Future investigations should be carried out considering a larger number of patients in each group and a longer supplementation time with silymarin. 

Fatigue, depression, and anxiety are common in RA patients, with a prevalence of 40–80% for fatigue [31] and up to 43 and 89% for depression and anxiety, respectively [43]. In our study, fatigue, depression, and anxiety increased under treatment with conventional DMARDs (control group) and underwent considerable reduction under supplementation with silymarin (intervention group). In various rodent models (neuroinflammation, depression), silymarin enhanced the levels of neurotransmitters (serotonin, dopamine, and norepinephrine) and brain-derived neurotrophic factor and reduced inflammation and oxidative stress in the hippocampus and frontal cortex. The antidepressant and anxiolytic properties of silymarin were confirmed in the forced swim, tail suspension, elevated plus maze, and open field tests [44]. 

Fatigue, depression, and anxiety were reported to correlate with disease severity and functional status in RA patients [33,45,46,47]. In our study, under supplementation with silymarin, all three comorbid conditions (fatigue, depression, and anxiety) associated well with the patients’ functional status. In addition, fatigue correlated well with the severity of disease activity. In patients receiving only conventional DMARDs (the control group), these correlations were weaker. These findings strongly support the ability of silymarin to potentiate the therapeutic efficacy and attenuate the side effects of DMARDs. 

The main strengths of this study include the recruitment of patients with active RA and long disease duration (>5 years), high patient adherence, and the presence of a control group. Our study also has some limitations. First, it is a single-center study. Furthermore, it enrolled a restricted number of patients and had a relatively short duration (8 weeks). These limitations may explain the lack of positive impact on the inflammatory markers ESR and CRP. 

## 5. Conclusions

Silymarin supplementation in patients with active RA under treatment with conventional DMARDs effectively improved disease severity and functional status (reduction in tender and swollen joints, morning stiffness, pain, disease activity and disability indices, and EULAR responses) and alleviated fatigue, anxiety, and depression. Our findings provide supportive evidence for the benefits of silymarin supplementation during conventional DMARD treatment. Further research (a multi-center trial enrolling a larger number of patients and having a longer duration) should be conducted to gain a better insight into the optimal posology of silymarin supplementation in RA. 

## Figures and Tables

**Table 1:** The baseline characteristics of the control and intervention groups.

Variable	Control Group (*n* = 61)	Intervention Group (*n* = 61)	*p* Value
Age (years) (95% CI)	58.90 ± 9.92 (56.36–61.44)	57.21 ± 8.19 (55.11–59.31)	0.308 ^a^
Gender (Female)	51 (49.5%)	52 (50.5%)	0.803 ^b^
Gender (Male)	10 (52.6%)	9 (47.4%)	
Residence (Rural)	29 (47.5%)	32 (52.5%)	0.587 ^b^
Residence (Urban)	32 (52.5%)	29 (47.5%)	
Height (cm) (95% CI)	165.67 ± 8.08 (163.60–167.74)	163.80 ± 7.43 (161.90–165.71)	0.186 ^a^
Weight (kg) (95% CI)	76.88 ± 15.87 (72.82–80.95)	73.95 ± 13.19 (70.57–77.33)	0.269 ^a^
BMI (kg/m^2^) (95% CI)	28.03 ± 5.51 (26.62–29.44)	27.53 ± 4.55 (26.36–28.69)	0.587 ^a^
Smoker	7 (50%)	7 (50%)	0.611 ^b^
Nonsmoker	55 (50%)	54 (50%)	
No. of comorbidities (95% CI)	2.36 ± 1.45 (1.99–2.73)	2.41 ± 1.38 (2.06–2.76)	0.848 ^a^
Disease duration (years) (95% CI)	6.95 ± 5.53 (5.53–8.37)	6.57 ± 5.08 (5.27–7.87)	0.696 ^a^
Disease stage (I/II)	28 (45.9%)	27 (44.3%)	0.856 ^b^
Disease stage (III/IV)	33 (54.1%)	34 (55.7%)	
Functional Capacity (I/II)	55 (90.2%)	56 (91.8%)	0.752 ^b^
Functional Capacity (III/IV)	6 (9.8%)	5 (8.2%)	
Autoantibodies			
RF (IU/mL) (95% CI)	87.24 ± 168.46 (42.12–132.35)	112.26 ± 220.35 (54.84–169.69)	0.497 ^a^
ANA (U/mL) (95% CI)	0.72 ± 1.23 (0.40–1.04)	0.63 ± 1.16 (0.33–0.94)	-
DNAds (U/mL) (95% CI)	14.18 ± 22.81 (8.23–20.12)	13.50 ± 21.01 (8.03–18.98)	-
ACPA (U/mL) (95% CI)	252.49 ± 386.11 (151.87–353.11)	238.06 ± 386.85 (137.28–338.85)	-

Age, height, weight, BMI (body mass index), number of comorbidities and disease duration, RF (rheumatoid factor), ANAs (antinuclear antibodies), anti-dsDNA (anti-(double-stranded)-DNA) antibodies, anti-CCP (anti-cyclic citrullinated peptides) antibodies—values are expressed as mean ± standard deviation and 95% confidence interval; gender, residence, smoking status, disease stage, and functional capacity—values express absolute frequencies and relative frequencies as percentages calculated according to the two groups (control, intervention) and the categories of each variable; ^a^ Independent *t* test; ^b^ Chi-Square test.

**Table 2:** Indicators of disease activity at baseline and week 8.

Outcome	Control Group	Intervention Group	*p* Value
**TJC28**			
Baseline (95% CI)	19.77 ± 5.89 (18.19–21.34)	20.07 ± 4.31 (18.94–21.19)	0.755 ^a^
Week 8 (95% CI)	21.38 ± 5.05 (20.03–22.73)	9.58 ± 3.68 (8.62–10.54)	0.000 ^a^
Change	−1.61 ± 2.94	10.49 ± 3.87	-
*p* value	0.000 ^b^	0.000 ^b^	-
**SJC28**			
Baseline (95% CI)	12.57 ± 4.63 (11.33–13.81)	12.69 ± 3.26 (11.84–13.54)	0.868 ^a^
Week 8 (*95% CI*)	13.57 ± 5.13 (12.20–14.95)	3.97 ± 2.42 (3.34–4.60)	0.000 ^a^
Change	−1.00 ± 2.87	8.73 ± 3.48	-
*p* value	0.012 ^b^	0.000 ^b^	-
**Morning stiffness (min.)**			
Baseline (95% CI) Week 8 (95% CI) Change *p* value	35.98 ± 21.20 (30.30–41.66) 38.93 ± 16.34 (34.55–43.30) −2.95 ± 11.94 0.070 ^b^	36.10 ± 20.97 (30.64–41.57) 27.97 ± 15.23 (23.99–31.93) 8.13 ± 19.67 0.002 ^b^	0.976 ^a^ 0.000 ^a^ - -
**Pain intensity (cm)**			
Baseline (95% CI)	7.61 ± 1.48 (7.21–8.00)	6.97 ± 1.29 (6.63–7.30)	0.015 ^a^
Week 8 (95% CI)	7.88 ± 1.25 (7.54–8.21)	3.56 ± 1.29 (3.22–3.89)	0.000 ^a^
Change	−0.27 ± 0.94	3.41 ± 1.12	-
*p* value	0.038 ^b^	0.000 ^b^	-
**PtGA**			
Baseline (95% CI)	7.61 ± 1.48 (7.21–8.00)	6.97 ± 1.29 (6.63–7.30)	0.015 ^a^
Week 8 (95% CI)	7.88 ± 1.25 (7.54–8.21)	3.56 ± 1.29 (3.22–3.89)	0.000 ^a^
Change	−0.27 ± 0.94	3.41 ± 1.12	-
*p* value	0.038 ^b^	0.000 ^b^	-
**PhGA**			
Baseline (95% CI)	7.61 ± 1.48 (7.21–8.00)	6.97 ± 1.29 (6.63–7.30)	0.015 ^a^
Week 8 (95% CI)	7.86 ± 1.29 (7.51–8.20)	3.56 ± 1.29 (3.22–3.89)	0.000 ^a^
Change	−0.25 ± 0.94	3.41 ± 1.12	-
*p* value	0.051 ^b^	0.001 ^b^	-

Values are expressed as mean ± standard deviation and 95% confidence interval; TJC28, tender joint count in 28 joints; SJC28, swollen joint count in 28 joints; PtGA, patient’s global assessment of disease activity; PhGA, physician’s global assessment of disease activity; ^a^ Independent *t* test; ^b^ Paired *t* test.

**Table 3:** Inflammatory markers at baseline and week 8.

Outcome	Control Group	Intervention Group	*p* Value
**ESR** (**mm/h)**			
Baseline (95% CI)	31.48 ± 27.05 (24.24–38.73)	24.10 ± 15.74 (20.00–28.20)	0.075 ^a^
Week 8 (95% CI)	28.45 ± 23.41 (22.18–34.72)	23.88 ± 15.07 (19.95–27.81)	0.220 ^a^
Change	3.04 ± 18.90	0.22 ± 9.32	-
*p* value	0.235 ^b^	0.857 ^b^	-
**CRP (mg/dL)**			
Baseline (95% CI) Week 8 (95% CI) Change *p* value	2.16 ± 4.45 (0.97–3.35) 1.44 ± 2.27 (0.84–2.05) 0.72 ± 4.06 0.191 ^b^	0.98 ± 1.32 (0.63–1.32) 1.06 ± 1.02 (0.80–1.33) −0.08 ± 1.35 0.632 ^b^	0.060 ^a^ 0.253 ^a^ - -

Values are expressed as mean ± standard deviation and 95% confidence interval; ESR, erythrocyte sedimentation rate; CRP, C reactive protein; ^a^ Independent *t* test; ^b^ Paired *t* test.

**Table 4:** Disease activity and disability indices at baseline and week 8.

Outcome	Control Group	Intervention Group	*p* Value
**DAS28 (ESR)**			
Baseline (95% CI)	6.68 ± 1.03 (6.41–6.96)	6.54 ± 0.67 (6.36–6.71)	0.378 ^a^
Week 12 (95% CI)	6.82 ± 0.97 (6.56–7.08)	4.82 ± 0.84 (4.60–5.04)	0.000 ^a^
Change	−0.14 ± 0.61	1.72 ± 0.61	-
*p* value	0.094 ^b^	0.000 ^b^	-
**DAS28 (CRP)**			
Baseline (95% CI)	6.25 ± 0.89 (6.01–6.49)	6.11 ± 0.63 (5.94–6.72)	0.311 ^a^
Week 12 (95% CI)	6.41 ± 0.83 (6.18–6.63)	4.42 ± 0.83 (4.21–4.64)	0.000 ^a^
Change	−0.15 ± 0.58	1.68 ± 0.70	-
*p* value	0.094 ^b^	0.000 ^b^	-
**SDAI**			
Baseline (95% CI)	50.43 ± 14.58 (46.52–54.33)	47.67 ± 8.51 (45.45–49.89)	0.222 ^a^
Week 12 (95% CI)	52.17 ± 12.68 (48.77–55.57)	21.44 ± 8.58 (19.20–23.67)	0.000 ^a^
Change	−1.74 ± 9.35	26.24 ± 8.20	-
*p* value	0.169 ^b^	0.000 ^b^	-
**CDAI**			
Baseline (95% CI)	48.27 ± 13.05 (44.77–51.76)	46.69 ± 8.30 (44.53–48.86)	0.445 ^a^
Week 12 (95% CI)	50.77 ± 11.93 (47.58–53.97)	20.37 ± 8.07 (18.27–22.47)	0.000 ^a^
Change	−2.51 ± 7.40	26.32 ± 7.88	-
*p* value	0.014 ^b^	0.000 ^b^	-
**HAQ-DI**			
Baseline (95% CI)	2.00 ± 0.45 (1.88–2.12)	1.89 ± 0.45 (1.78–2.02)	0.212 ^a^
Week 12 (95% CI)	2.17 ± 0.39 (2.06–2.27)	1.18 ± 0.36 (1.08–1.27)	0.000 ^a^
Change	−0.17 ± 0.25	0.72 ± 0.31	-
*p* value	0.000 ^b^	0.000 ^b^	-

Values are expressed as mean ± standard deviation and 95% confidence interval; DAS28-ESR, disease activity score in 28 joints calculated with erythrocyte sedimentation rate; DAS28-CRP, disease activity score in 28 joints calculated with C reactive protein; SDAI, simplified disease activity index; CDAI, clinical disease activity index; HAQ-DI, health assessment questionnaire-disability index; ^a^ Independent *t* test; ^b^ Paired *t* test.

**Table 5:** Fatigue, depression, and anxiety scores at baseline and week 8.

Outcome	Control Group	Intervention Group	*p* Value
**VAS-F**			
Baseline (95% CI)	71.61 ± 12.90 (68.15–75.06)	70.76 ± 11.88 (67.67–73.86)	0.716 ^a^
Week 8 (95% CI)	75.36 ± 10.61 (72.52–78.20)	37.80 ± 10.39 (35.09–40.50)	0.000 ^a^
Change	−3.75 ± 11.25	32.97 ± 13.55	-
*p* value	0.016 ^b^	0.000 ^b^	-
**BDI-II**			
Baseline (95% CI)	19.48 ± 6.78 (17.67–21.30)	18.03 ± 5.64 (16.56–19.50)	0.215 ^a^
Week 8 (95% CI)	21.21 ± 5.15 (19.83–22.59)	8.90 ± 3.48 (7.99–9.81)	0.000 ^a^
Change	−1.73 ± 3.62	9.14 ± 3.73	-
*p* value	0.001 ^b^	0.000 ^b^	-
**GAD-7**			
Baseline (95% CI)	11.05 ± 3.59 (10.09–12.02)	10.69 ± 4.17 (9.61–11.78)	0.623 ^a^
Week 8 (95% CI)	12.11 ± 2.79 (11.36–12.86)	3.54 ± 2.01 (3.02–4.07)	0.000 ^a^
Change	−1.05 ± 2.22	7.15 ± 3.41	-
*p* value	0.001 ^b^	0.000 ^b^	-

Values are expressed as mean ± standard deviation and 95% confidence interval; VAS-F, visual analogue scale-fatigue; BDI-II, Beck depression inventory II; GAD-7, generalized anxiety disorder-7; ^a^ Independent *t* test; ^b^ Paired *t* test.

**Table 6:** Pearson’s correlation coefficients between disease activity and disability indices and levels of fatigue, depression, and anxiety at week 8.

	Group	DAS28- ESR	DAS28- CRP	SDAI	CDAI	HAQ-DI	VAS-F	BDI-II	GAD-7
DAS28-ESR	I		0.836	0.858	0.838	0.426	0.466	0.180	0.159
			(0.000)	(0.000)	(0.000)	(0.001)	(0.000)	(0.172)	(0.229)
	C		0.824	0.757	0.667	0.305	0.316	0.250	0.243
			(0.000)	(0.000)	(0.000)	(0.022)	(0.018)	(0.063)	(0.071)
DAS28-CRP	I			0.866	0.824	0.401	0.506	0.251	0.126
				(0.000)	(0.000)	(0.002)	(0.000)	(0.055)	(0.342)
	C			0.790	0.685	0.210	0.306	0.328	0.211
				(0.000)	(0.000)	(0.121)	(0.022)	(0.014)	(0.118)
SDAI	I				0.987	0.353	0.494	0.200	0.159
					(0.000)	(0.006)	(0.000)	(0.128)	(0.229)
	C				0.908	0.279	0.225	0.165	0.162
					(0.000)	(0.038)	(0.096)	(0.225)	(0.234)
CDAI	I					0.357	0.505	0.185	0.164
						(0.005)	(0.000)	(0.161)	(0.213)
	C					0.294	0.191	0.097	0.193
						(0.028)	(0.159)	(0.476)	(0.154)
HAQ-DI	I						0.387	0.442	0.437
							(0.002)	(0.000)	(0.001)
	C						0.271	0.288	0.211
							(0.044)	(0.032)	(0.118)
VAS-F	I							0.423	0.419
								(0.001)	(0.001)
	C							0.394	0.389
								(0.003)	(0.003)
BDI-II	I								0.651
									(0.000)
	C								0.618
									(0.000)

I: intervention group; C: control group; DAS28-ESR: disease activity score in 28 joints calculated with erythrocyte sedimentation rate; DAS28-CRP: disease activity score in 28 joints calculated with C reactive protein; SDAI: simplified disease activity index; CDAI: clinical disease activity index; HAQ-DI: health assessment questionnaire-disability index; BDI-II: Beck depression inventory II; GAD-7: generalized anxiety scale-7; VAS-F: visual analogue scale-fatigue.

## Data Availability

The original contributions presented in this study are included in the article; further inquiries can be directed to the corresponding authors.
